# Regional myocardial microvascular dysfunction in cardiac amyloid light-chain amyloidosis: assessment with 3T cardiovascular magnetic resonance

**DOI:** 10.1186/s12968-016-0240-7

**Published:** 2016-04-06

**Authors:** Rui Li, Zhi-gang Yang, Lin-yi Wen, Xi Liu, Hua-yan Xu, Qin Zhang, Ying-kun Guo

**Affiliations:** Department of Radiology, West China Hospital, Sichuan University, 37# Guo Xue Xiang, Chengdu, Sichuan 610041 China; National Key Laboratory of Biotherapy, West China Hospital, Sichuan University, 17# Section 3 South Renmin Road, Chengdu, Sichuan 610041 China; Department of Radiology, West China Second University Hospital, Sichuan University, 20# Section 3 South Renmin Road, Chengdu, Sichuan 610041 China; Department of Radiology, Affiliated Hospital of North Sichuan Medical College, 63# Wenhua Road, Shunqing District, Nanchong, Sichuan 637000 China

**Keywords:** Amyloid light-chain cardiac amyloidosis, Cardiovascular magnetic resonance, Perfusion imaging, Left ventricular function, Coronary microvascular function

## Abstract

**Background:**

Coronary microvascular dysfunction is highly prevalent in patients with amyloid light-chain (AL) cardiac amyloidosis (AL-CA). The aim of this study was to clarify the feasibility of first-pass perfusion imaging using 3 T cardiovascular magnetic resonance (CMR) for evaluating the difference in left ventricular (LV) regional myocardial microvascular function among normal subjects and in patients with AL-CA. The amyloidosis patients were classified into those with impaired systolic function [LV ejection fraction (LVEF) < 50 %] and those with preserved systolic function.

**Methods:**

In total, 32 patients with biopsy-proven AL-CA, including 11 AL-CA patients with systolic dysfunction, 21 AL-CA patients with preserved systolic function, and 25 healthy subjects, underwent CMR examination. LV regional myocardial perfusion parameters included upslope, time to maximum signal intensity (TTM) and max signal intensity (MaxSI) were compared among the three patient groups. Receiver operating characteristic analysis was performed to determine whether perfusion parameters could be used in discriminating regional myocardial microvascularity between AL-CA patients and normal subjects.

**Results:**

The patients with AL-CA had significantly reduced first-pass perfusion upslope and MaxSI, and increased TTM compared with the normal subjects (all *P* < 0.01). Compared with the patients with AL-CA and preserved LVEF, the patients with AL-CA and impaired systolic function had a longer TTM in the basal (47.05 ± 16.59 vs. 39.68 ± 19.11; *P* = 0.002) and mid-ventricular (44.61 ± 16.34 vs. 37.74 ± 18.25; *P* = 0.002) segments; lower upslope in the basal (2.41 ± 1.32 vs. 3.60 ± 1.68; *P* < 0.0001), mid-ventricular (2.82 ± 1.34 vs. 4.15 ± 2.02; *P* < 0.0001), and apical (3.71 ± 1.38 vs. 4.97 ± 2.55; *P* = 0.004) segments; and lower MaxSI (31.67 ± 15.23 vs. 37.96 ± 11.15; *P* < 0.0001) in the basal segment. The ROC curve analysis revealed that the first-pass upslope, TTM, and MaxSI may be used as indicators for differentiating microcirculation between AL-CA patients with preserved or impaired systolic function and normal subjects.

**Conclusions:**

The differences in LV regional myocardial microvascular function among normal subjects, AL-CA patients with systolic dysfunction, and AL-CA patients with preserved systolic function can be monitored using first-pass perfusion CMR.

## Background

Amyloidosis is a rare disorder characterized by the extracellular deposition of pathological insoluble proteins in multiple organs, including the heart [1, 2]. Amyloid light-chain (AL) amyloidosis, in which amyloid fibrils are derived from the monoclonal immunoglobulin light chain, is one the most common types of amyloidosis [3]. Cardiac involvement, termed cardiac amyloidosis (CA), is observed in approximately 50 % patients with systemic amyloidosis and is also the major cause of death in patients with AL amyloidosis [4, 5]. AL-CA has a poor prognosis, particularly in patients with impaired systolic function and abnormal wall thickening. The left ventricular ejection fraction (LVEF), which is widely used as an index of systolic function in clinical practice, is considered an independent predictor of cardiac mortality in AL amyloidosis [3, 4].

Necroscopy examinations have confirmed that amyloid infiltration in the microvascular system may cause coronary microcirculation disorder, with patients presenting with symptoms of angina and signs of ischemia [6–8]. In addition, increased wall thickness, an important morphologic feature of CA, may affect myocardial microvascular perfusion due to vascular rarefaction and compression [9].

As amyloid infiltration in the cardiac microvascular system is prevalent in patients with CA and is potentially associated with LV systolic function and wall thickening [3, 9], it is essential to evaluate coronary microvascular perfusion in patients with AL-CA, particularly in those with systolic dysfunction. Clinically, contrast-enhanced cardiovascular magnetic resonance (CE-CMR) is regarded as a well-established method for the assessment of cardiac morphology, function, and tissue characteristics [10, 11]. High field 3 T CMR improves the signal to noise ratio, contrast to noise ratio, and first-pass myocardial perfusion CMR [12, 13]. Higher levels of accuracy, sensitivity, specificity and reproducibility can be achieved by using CMR perfusion parameters, including slope, TTM, and MaxSI [12, 14, 15]. These parameters, derived from the CMR signal intensity-time curve and reflected myocardial perfusion reserve, are associated with coronary microvascular function [16]. By using a first-pass myocardial perfusion technique, CMR can be used to monitor myocardial microvascular dysfunction in hypertrophic and dilated cardiomyopathy in a non-invasive manner [17, 18]. Additional, CE-CMR imaging allows the simultaneous assessment of both myocardial perfusion and function in a single investigation. However, to the best of our knowledge, few studies have investigated myocardial microvascular dysfunction in patients with AL-CA using CMR. Thus, the aim of this study was to assess regional myocardial perfusion and function in AL-CA patients using 3.0-T CE-CMR imaging and to determine whether these perfusion parameters may assist in discriminating between the microcirculation of AL-CA patients with or without LV systolic dysfunction and that of normal subjects.

## Methods

### Patients

The institutional ethics committee of our hospital approved this study, and written informed consent was obtained from each participant prior to the investigation. Between September 2013 and March 2015, 38 patients with biopsy-proven AL amyloidosis were enrolled in the study, according to the following inclusion criteria: (a) AL amyloidosis, initially diagnosed in extracardiac tissue using congo red and immunohistochemical staining and (b) cardiac involvement, confirmed according to echocardiographic criteria [19]. The exclusion criteria included (a) associated diseases that may cause myocardium hypertrophy and/or coronary mircovascular dysfunction, such as hypertrophic cardiomyopathy, diabetes, hypertension, coronary artery disease, and arrhythmia and (b) non-interpretable MR image quality, unsuitable to enable diagnosis. In total, 32 AL-CA patients remained following the exclusion of six patients who met the aforementioned exclusion criteria. The mean patient age was 60 years (range, 38–75 years). In total, 15 of the 32 patients (47 %) were women. During the same period, 25 normal controls (13 males and 12 females; mean age, 37.68 ± 11.94 years; range, 17–58 years) underwent CMR on initial clinical suspicion of cardiovascular disease but were confirmed as healthy subjects by clinical evidence and served as a control group. The exclusion criteria included chronic disease, family history of cardiovascular disease, diabetes, hypertension (>140/90 mmHg), and arrhythmia. Prior to CMR, routine clinical echocardiography was performed in all subjects using two-dimensional transthoracic echocardiography (TEE) with a multiplanar 3.5-MHz probe (IE33; Philips Medical Systems, Andover, MA, USA), according to the guidelines of the American Society of Echocardiography [20]. Endomyocardial biopsy was not performed in our study due to its invasive nature and the severity of its potential complications [21]. Thus, the patients were diagnosed with CA via extracardiac tissue biopsy, which was obtained through specimens of kidney (*n =* 6; 18.8 %), bone marrow (*n =* 27; 84.4 %), liver (*n =* 1; 3.1 %), fat (*n =* 6; 37.5 %), rectum (*n =* 1; 3.1 %), skin (*n =* 5; 15.6 %), and tongue (*n =* 1; 3.1 %) and typical echocardiographic findings, including a mean wall thickness of the ventricular wall and/or interventricular septum measuring >12 mm in diastole [19].

### CMR

All patients were examined in the supine position using a 3 T whole-body scanner (Trio Tim; Siemens Medical Solutions, Erlangen, Germany) at least 3 days following ECG examination. A dedicated two-element cardiac-phased array coil was used for signal detection. The manufacturer’s standard ECG-triggering device and the breath-hold technique were used to monitor the individuals’ ECG values and breathing, respectively. Following a survey scan, cine images were acquired in 2-chamber, 3-chamber, and 4-chamber views using a TrueFISP sequence (TR/TE, 44.7/1.33 ms; flip angle, 50°; field of view, 290 mm × 373 mm; matrix size 146 mm × 224 mm; slice thickness, 8 mm). Subsequently, gadobenate dimeglumine (MultiHance; 0.5 mmol/ml; Bracco, Milan, Italy) was intravenously injected using an automated injector (Stellant, MEDRAD, Indianola, PA, USA) at a dose of 0.1 ml/kg body weight and a flow rate of 2.5–3.0 ml/s. In addition, a 20 ml saline flush was injected immediately following contrast at a rate of 3.0 ml/s. Rest regional perfusion images were acquired in three standard short-axis slices (apical, middle, and basal) and in one slice of the 4-chamber view using an inversion-recovery prepared echo-planar imaging sequence (repetition time, 200 ms; echo time, 1.1 ms; inversion time, 90.0 ms; flip angle, 10°; field of view, 270 mm × 360 mm; acquisition matrix, 106 × 192; slice thickness, 8 mm). Each set of first-pass perfusion images was completed in 80 cardiac cycles.

### CMR data analysis

**Fig. 1:**
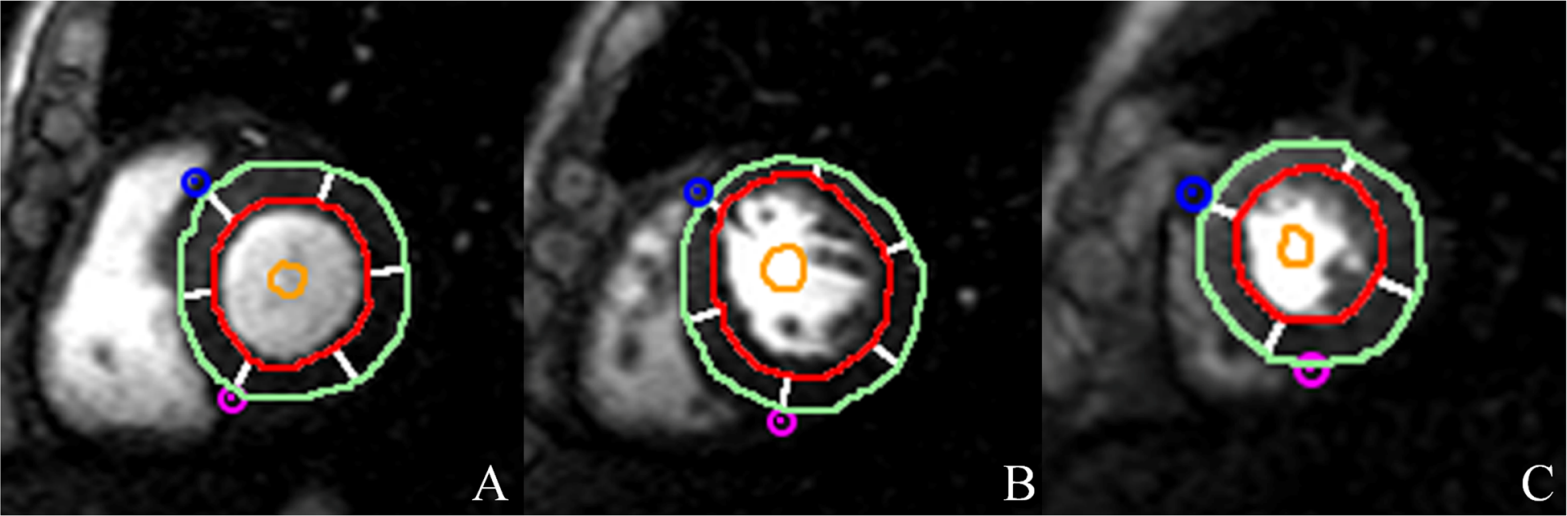
AHA bull’s eye model according to standard segmentation for regional analysis. Based on this model, segmentation of the LV on myocardial first-pass perfusion images included the following: (**a**) Basal segments; (**b**) Mid-ventricular segments; and (**c**) Apex segments. LV wall end-diastolic thickness was also determined using this model

**Fig. 2:**
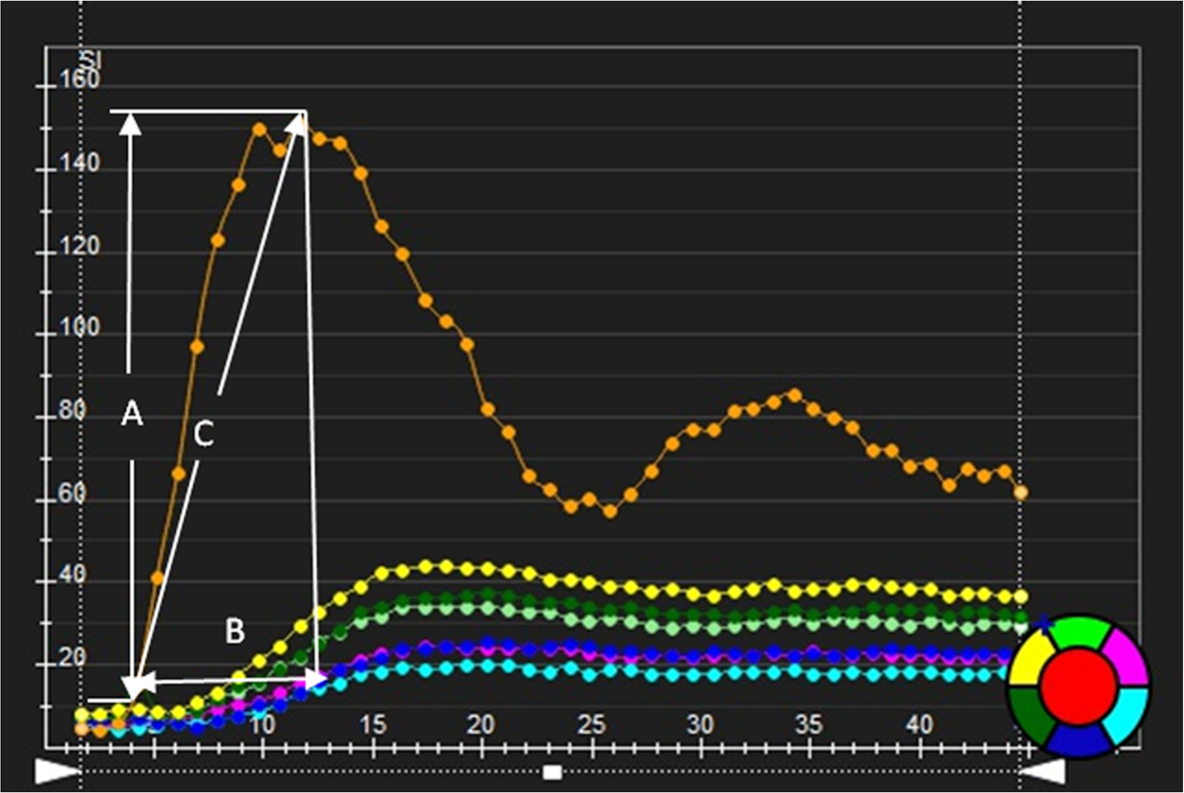
The signal intensity-time curve derived from myocardial perfusion images. The first-pass perfusion values including MaxSI (**a**), time to max signal intensity (**b**) and first-pass perfusion upslope (**c**) were automatically obtained

Image analysis was performed offline using commercial software (cvi^42^; Circle Cardiovascular Imaging, Inc., Calgary, Canada). Endocardial and epicardial traces were performed manually by two experienced observers (Wen LY and Li R) in the serial short-axis slices at the end-diastolic and end-systolic phases. The global cardiac function, including LV end-diastolic volume (EDV), end-systolic volume (ESV), and LV ejection fraction (LVEF) were computed using a modified Simpson’s rule. For regional analysis, a 16-segment mode (Bull’s eye plot), according to AHA standard segmentation, was constructed on the basis of analysis of the long- and short-axis slices (apical, mid-ventricular, and basal; Fig. 1), which included four apical segments (septum, anterior, lateral, and inferior), and six mid-ventricular and basal segments (inferior septum, anterior septum, anterior, anterolateral, inferolateral, and inferior). Morphologically, the average LV end-diastolic and -systolic wall thickness for each segments were measured, and the myocardial wall thickening, defined as the variation in thickness between diastole to systole, expressed as a percentage (Wth) were calculated using the centerline method [22]. For evaluation of the LV regional perfusion, the endocardial, epicardial, and blood pool contours of all three sets of first-pass perfusion images (basal, mid-ventricular, and apical) were delineated manually, and the myocardial signal intensity-time curve was processed using the aforementioned software. First-pass parameters including upslope, time to maximum signal intensity (TTM) and max signal intensity (MaxSI) were consequently obtained from the myocardial signal intensity-time curve (Fig. 2).

### Statistical analysis

All statistical analyses were performed using SPSS software (version 19.0 for Windows; SPSS, Chicago, IL, USA). The results are expressed as the mean ± standard deviation (SD). Continuous variables were compared using independent Student’s *t*-test or one-way analysis of variance (ANOVA). A Mann–Whitney *U* test was used to compare the regional myocardial perfusion and functional parameters among the normal subjects and the AL-CA patients with or without systolic dysfunction using Bonferroni’s correction for multi-group comparisons. If a significant difference was confirmed, receiver operating characteristic (ROC) analysis was performed to determine whether the cut-off values of regional cardiac perfusion can be used to differentiate myocardial microvascular function between AL-CA patients and normal subjects. Spearman’s rank correlations analysis was performed to examine the correlation between regional myocardial perfusion, wall thickness, and wall thickening. *P* < 0.05 was considered to indicate a statistically significant difference.

## Results

### Baseline characteristics

The baseline characteristics the AL-CA patients and normal subjects are presented in Table 1. Of the 32 cardiac patients, 11 (34.4 %) presented with impaired systolic function (LVEF < 50 %). Half of the AL-CA patients had a history of New York Heart Association functional class ≥ III heart failure. The LVEDV of the AL-CA patients with preserved LVEF was lower than that of the normal healthy controls (*P* = 0.013). The AL-CA patients with impaired LVEF had a significantly higher LVESV and lower SV than that of the normal subjects and the AL-CA patients with preserved LVEF (all *P* < 0.001). However, no statistically significant differences were observed between the patients with preserved LVEF and the normal subjects in LVESV (47.45 ± 12.97 vs. 52.62 ± 14.01 ml; *P* = 0.693) or LVEF (56.62 ± 9.43 vs. 60.18 ± 4.30 %; *P* = 0.059). The LV mass in the AL-CA patients were markedly increased as compared with that in the normal subjects (both *P* < 0.0001); however, there was no significant difference between the AL-CA patients with LV systolic dysfunction and those without LV systolic dysfunction (143.55 ± 42.64 vs. 127.14 ± 32.29 g; *P* = 0.484). After CMR and echocardiography, fifteen (46.9 %) patients received chemotherapy whereas seventeen (53.1 %) did not, and the chemotherapy regimen was listed in Table 2.

**Table 1:** Baseline Differences Between Cardiac Normal healthy, AL-CA patients with or without LV systolic dysfunction

	Normal subjects (*n =* 25)	CA without SD (*n =* 21)	CA with SD (*n =* 11)
Age (Y)	37.68 ± 11.94	60.10 ± 7.85^*^	59.82 ± 12.14^*^
Male	13 (52)	11 (52)	6 (55)
NYHA functional class			
I	-	2 (9)	0 (0)
II	-	10 (48)	4 (36)
III	-	9 (43)	5 (46)
IV	-	0 (0)	2 (18)
Echocardiography			
Septal thickness (mm)	8.73 ± 1.55	15.29 ± 2.83*	17.27 ± 2.57*
E/A	1.2 ± 0.2	1.7 ± 0.6*	2.4 ± 0.8*^§^
E/E’	11.11 ± 2.21	17.14 ± 4.33*	20.82 ± 5.69*^§^
Cine-CMR			
LVEDV (ml)	133.78 ± 25.43	111.06 ± 26.84*	118. 20 ± 24.47
LVESV (ml)	52.62 ± 14.01	47.45 ± 12.97	71.95 ± 17.71*^§^
LV SV (ml)	81.18 ± 15.29	63.60 ± 23.44*	46.25 ± 10.78*^§^
LVEF (%)	60.18 ± 4.30	56.62 ± 9.43	39.28 ± 6.39*^§^
LV mass (g)	85.32 ± 23.32	127.14 ± 32.29*	143.55 ± 42.64*
Pericardial effusion	-	12 (57)	4 (36)
Pleural effusion	-	13 (67)	7 (63)

*Notes:* The values are the mean ± SD, Numbers in the brackets are percentages. ^*^ *P* < 0.017 versus normal group; ^§^ *P* < 0.0017 versus CA with preserved LVEF. AL-CA = light-chain amyloid cardiac amyloidosis; SD = systolic dysfunction; LV = left ventricular; EDV = end diastolic volume; ESV = end systolic volume; EF = ejection fraction; SV = stroke volume

**Table 2:** Chemotherapeutic agents used

Chemotherapy regimen	No. of subjects
Cyclophosphamide, thalidomide, dexamethasone	4
Cyclophosphamide, thalidomide, dexamethasone/prednisone	1
Bortezomib, dexamethasone, cyclophosphamide	2
Cyclophosphamide, prednisone/mesna	1
Cyclophosphamide, prednisone	2
Cyclophosphamide, prednisone/dexamethasone	1
Cyclophosphamide, dexamethasone, vindesine, epirubicin	1
Cyclophosphamide, dexamethasone	1
Thalidomide, dexamethasone	1
Bortezomib, prednisone	1
None	17

### LV wall functional analysis

The AL-CA patients had significantly thicker EDWT and ESWT and reduced Wth compared with the normal controls (all *P* < 0.05; Table 3). Compared with the AL-CA patients with preserved LVEF, the AL-CA patients with LV systolic dysfunction presented with reduced Wth (*P* < 0.0001) and increased EDWT (*P* = 0.014). However, there was no significant difference in ESWT between the AL-CA patients with LV systolic dysfunction and those without LV systolic dysfunction. Using the Bull’s eye plot, baso-apical gradients of decreased EDWT and ESWT and increased Wth were observed in the AL-CA patients. The average differences in EDWT, ESWT, and Wth in the basal, mid-ventricular, and apical segments between the AL-CA patients with or without LV systolic dysfunction were compared (Table 4). The AL-CA patients with LV systolic dysfunction presented with lower Wth in the basal and mid-ventricular segments as compared with the AL-CA patients with preserved LVEF (27.70 ± 23.11 vs. 48.91 ± 39.54; *P* = 0.001 and 65.00 ± 44.18 vs. 86.30 ± 52.67; *P* = 0.007, respectively) and thicker EDWT in the basal segment (13.90 ± 3.11 vs. 12.85 ± 3.76; *P* = 0.02). However, no significant differences in ESWT were observed between the AL-CA patients with or without LV systolic dysfunction in any of the three segments.

**Table 3:** First-pass perfusion and regional function data of all study groups

	Normal controls (*n =* 25)	CA without SD (*n =* 21)	CA with SD (*n =* 11)
Upslope	6.64 ± 2.03	4.15 ± 2.11*	2.89 ± 1.43*^§^
TTM (sec)	20.35 ± 13.74	39.00 ± 18.60*	45.81 ± 16.25^*§^
MaxSI	52.74 ± 12.10	42.08 ± 12.79*	38.21 ± 18.34^*§^
EDWT (mm)	6.92 ± 1.99	10.28 ± 3.87*	11.06 ± 3.72*^§^
ESWT(mm)	12.71 ± 3.51	16.74 ± 3.59*	16.29 ± 3.91*
Wth(%)	93.18 ± 62.07	77.97 ± 53.05*	58.64 ± 50.59*^§^

*Notes:* The values are the mean ± SD, ^*^ *P* < 0.017 versus normal group; ^§^ *P* < 0.017 versus CA without systolic function; SD = systolic dysfunction; TTM = time to max; EDWT = end-diastole wall thickness; ESWT = end-systole wall thickness; Wth = wall thickening

**Table 4:** Regional comparison of first perfusion and function parameters between AL-CA subject with or without LV systolic dysfunction

	Basal			Mid-ventricular			apex		
	Normal	CA without SD	CA with SD	Normal	CA without SD	CA with SD	Normal	CA without SD	CA with SD
Upslope	6.52 ± 1.85	3.60 ± 1.68^*^	2.41 ± 1.32^*§^	6.57 ± 2.07	4.15 ± 2.02^*^	2.82 ± 1.34^*§^	6.95 ± 2.22	4.97 ± 2.55^*^	3.71 ± 1.38^*§^
TTM (sec)	19.51 ± 9.68	39.68 ± 19.11^*^	47.05 ± 16.59^*§^	19.78 ± 16.94	37.74 ± 18.25^*^	44.61 ± 16.34^*§^	22.47 ± 13.47	39.88 ± 18.46^*^	45.75 ± 15.83^*^
MaxSI	51.17 ± 10.86	37.96 ± 11.15^*^	31.67 ± 15.23^*§^	52.96 ± 12.20	41.61 ± 12.18^*^	38.07 ± 18.24^*^	53.96 ± 13.62	48.95 ± 13.24^*^	48.21 ± 18.63^*^
EDWT (mm)	8.36 ± 1.88	12.85 ± 3.76^*^	13.90 ± 3.11^*^	6.71 ± 1.44	9.82 ± 3.08^*^	10.41 ± 3.02^*^	5.08 ± 1.00	7.09 ± 2.04^*^	7.77 ± 1.96^*^
ESWT (mm)	13.86 ± 3.99	18.00 ± 3.03^*^	17.43 ± 3.81^*^	12.86 ± 2.80	17.14 ± 3.40^*^	16.39 ± 3.97^*^	10.75 ± 2.84	14.27 ± 3.45^*^	14.44 ± 3.29^*^
Wth(%)	72.14 ± 59.86	48.91 ± 39.54^*^	27.70 ± 23.11^*§^	97.47 ± 53.27	86.30 ± 52.67	65.00 ± 44.18^*§^	118.29 ± 67.79	109.06 ± 49.61	95.51 ± 61.16^*^

*Notes:* The values are the mean ± SD, other abbreviations are the same as in Tables 1 and 2. ^*^ *P* < 0.017 versus normal group; ^§^ *P* < 0.017 versus CA without systolic dysfunction

### First pass perfusion analysis

The first-pass perfusion parameters of all subjects are shown in Table 3. Using the Bull’s eye mode with regional wall functional analysis, the average first perfusion parameters of the basal, mid-ventricular, and apical segments were calculated (Table 4). Gradual increases in upslope and MaxSI, from base to apex, were observed in the AL-CA patients, regardless of LVEF. Compared with the normal controls, the upslope and MaxSI of the AL-CA patients were significantly reduced, and the TTM was significantly increased (all *P* < 0.01). The AL-CA patients with LV systolic dysfunction exhibited a shorter upslope in the three segments (all *P* < 0.01), reduced MaxSI in the basal segment, and longer TTM in the basal and mid-ventricular segments (all *P* < 0.01) as compared with the AL-CA patients without LV systolic dysfunction.

### Correlation between regional first-perfusion, wall thickness, and wall thickening

A significant and positive correlation was demonstrated between the first-pass upslope and Wth (Spearman’s rank = 0.258; *P* < 0.0001) and between the MaxSI and Wth (Spearman’s rank = 0.338; *P* < 0.0001). A negative correlation was found between the first-pass perfusion upslope and EDWT (Spearman’s rank = −0.431; *P* < 0.0001) and ESWT (Spearman’s rank = −0.355; *P* < 0.0001) and between the MaxSI and EDWT (Spearman’s rank = −0.458; *P* < 0.0001) and ESWT (Spearman’s rank = −0.288; *P* < 0.0001).

### ROC curve analysis

**Fig. 3:**
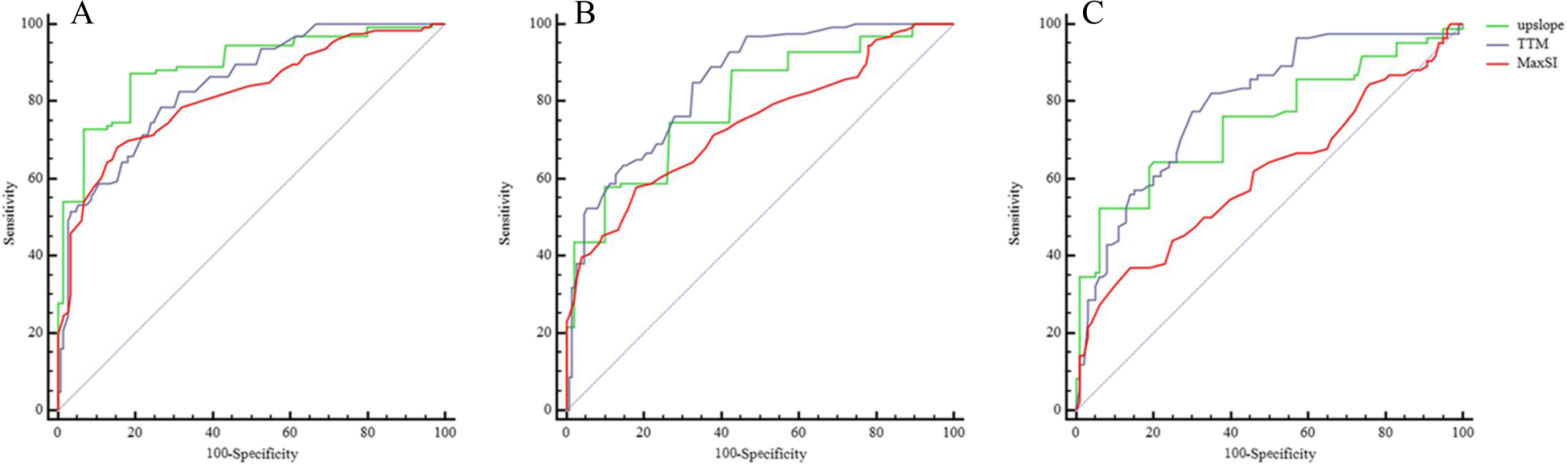
Receiver-operating characteristic analysis (ROC) between AL-CA patients with preserved LV systolic function and normal controls. By using the cut-off values of upslope (Green), TTM (Blue) and MaxSI (Red), the first-pass perfusion MR could discriminate the myocardial microvascularity of AL-CA patients with preserved LV systolic function from that of normal controls in the basal (**a**), mid-ventricular (**b**) and apex (**c**) segments

**Fig. 4:**
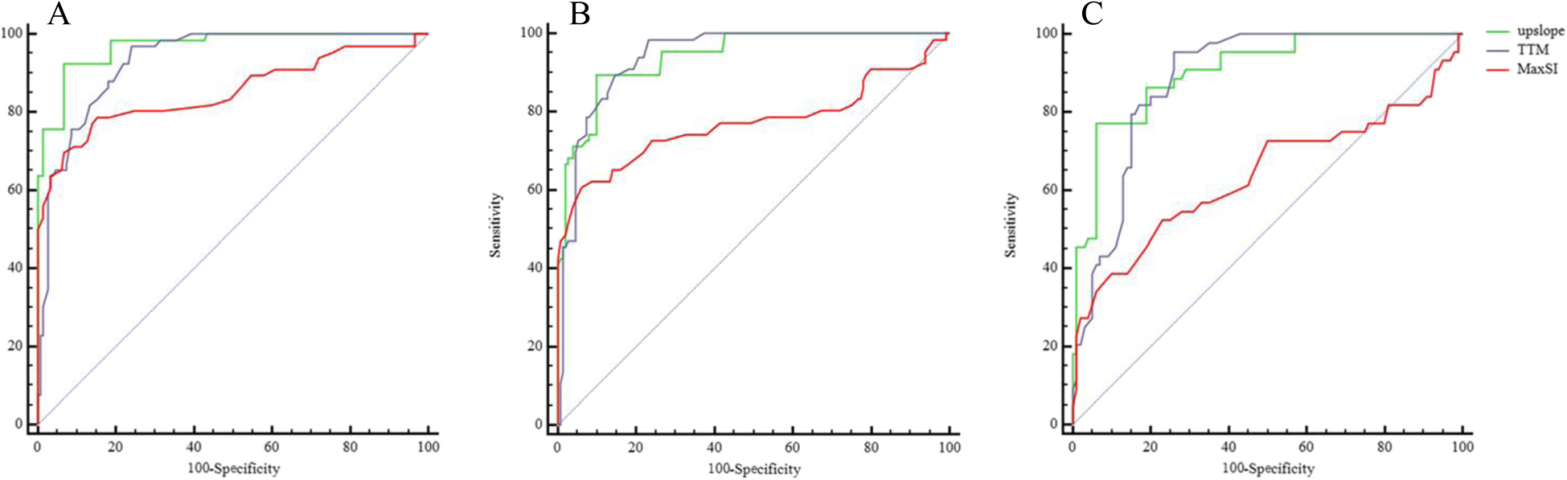
Receiver-operating characteristic (ROC) analysis between AL-CA patients with impaired LV systolic function and normal controls. The use of cut-off values of upslope (Green), TTM (Blue) and MaxSI (Red) could discriminate the microcirculation of AL-CA patients with impaired LV systolic function from that of normal controls in the basal (**a**), mid-ventricular (**b**) and apex (**c**) segments

Following ROC analysis in the basal, mid-ventricular, and apical segments, we found that the cut-off values for the first-pass upslope, TTM, and MaxSI assisted in discriminating between the microvascularity of AL-CA patients with preserved or impaired LVEF and normal controls (Figs. 3 and 4). The area under the ROC curve, and the sensitivity and specificity of the LV regional first by-pass perfusion data used for the discrimination regional myocardial microvascularity between AL-CA patients with or without LV systolic dysfunction and normal controls are summarized in Tables 5 and 6.

**Table 5:** ROC analysis of first-pass perfusion for detecting microvascular dysfunction between AL-CA patients with preserved systolic function and normal controls

	Cutoff	AUC	Sensitivity (%) (95 % CI)	Specificity (%) (95 % CI)
Basal segment				
Upslope	5	0.888	87 (82–93)	81 (74–87)
TTM	20.8	0.843	79 (70–85)	73 (66–80)
MaxSI	42	0.816	68 (59–76)	85 (78–90)
Mid-ventricular segment				
Upslope	4	0.794	58 (49–67)	90 (84–94)
TTM	19.9	0.848	85 (78–91)	67 (59–75)
MaxSI	42	0.741	58 (49–67)	82 (75–88)
Apical segment				
Upslope	4	0.747	52 (41–63)	94(87–98)
TTM	21.87	0.795	77 (67–86)	70 (60–79)
MaxSI	41	0.610	37 (27–48)	86 (78–92)

*Notes:* AUC = area under the ROC curve. The abbreviations are the same as in Tables 1 and 2

**Table 6:** ROC analysis of first-pass perfusion for detecting microvascular dysfunction between AL-CA patients with impaired systolic function and normal controls

	Cutoff	AUC	Sensitivity (%) (95 % CI)	Specificity (%) (95 % CI)
Basal segment				
Upslope	4.1	0.969	92 (83–98)	93 (88–97)
TTM	21.9	0.932	97 (90–100)	76 (68–83)
MaxSI	42	0.852	79 (67–88)	85 (78–90)
Mid-ventricular segment				
Upslope	4	0.941	89 (79–96)	90 (84–94)
TTM	21.68	0.941	98 (92–100)	77 (69–83)
MaxSI	36	0.772	61 (48–72)	94 (89–97)
Apical segment				
Upslope	4.57	0.906	77(62–88)	94 (87–98)
TTM	23	0.886	95 (85–99)	74 (64–82)
MaxSI	43	0.638	52 (37–68)	77 (68–85)

*Notes:* AUC = area under the ROC curve. The abbreviations are the same as in Tables 1 and 2

## Discussion

In this study, we assessed the use of 3 T CMR to provide information on regional myocardial microcirculation and wall abnormalities in AL-CA patients. We found that the TTM, EDWT, and ESWT were increased and the first-pass perfusion slope, MaxSI, and Wth were decreased significantly in the AL-CA patients compared with the normal controls. Furthermore, coronary microvascular dysfunction was significantly correlated with LV wall thickness and wall thickening, which is consistent with a previous report [9]. These findings suggested that myocardial perfusion in AL-CA patients is poorer than that in healthy individuals due to coronary microcirculation disorder. The increased wall thickness and abnormal wall thickening may also contribute to myocardial perfusion dysfunction.

Coronary microvascular dysfunction is common in patients with AL amyloidosis. Symptoms of angina and ischemia, impaired vasodilatation, and minimal coronary vascular resistance have been described in CA patients without coronary artery disease [9, 23–25]. The amyloid deposition leading to coronary microvascular dysfunction in CA patients may be sustained by several pathogenetic mechanisms, including structural (amyloid infiltrated the vascular wall causing wall thickening and luminal obstruction), functional (autonomic and endothelial) and extravascular (perivascular and interstitial amyloid deposits leading to extramural compression and decreased diastolic perfusion time) [9, 26]. In addition, the mechanisms of coronary microvascular dysfunction in other non-ischemic cardiomyopathies, including small-vessel vasculitides, coronary microvascular remodeling, interstitial fibrosis, increased LV end-diastolic pressures and LV myocardial mass, and decreased capillary density may assist in explaining coronary microcirculation disorder in CA [27, 28].

A gradient of basoapically decreasing/increasing slope, MaxSI, and Wth gradient were also observed in the AL-CA patients. This basoapical gradient also reflecting underlying myocardial wall stress in the left ventricle, are in accordance with other CMR findings which showed greater Late Gadolinium Enhancement (LGE) area at the base compared with the apex segments, and echocardiographic studies using strain analysis, which reported reduced basal systolic longitudinal and radial strain in CA [29–32].

Another finding in our study was that the AL-CA patients with impaired LVEF were associated with more marked regional LV microvascular disorder as compared with the AL-CA patients with preserved LVEF. As an independent predictor of cardiac mortality in AL amyloidosis patients, systolic dysfunction tends to occur in advanced stages of the disease and is often associated with poor outcome [4, 33, 34]. AL-CA patients with impairment of systolic function have been considered inappropriate for autologous stem cell transplantation, which is generally accepted as a therapeutic approach to improve the survival rates and quality of life in AL amyloidosis [35–37]. Thus, early identification of the systolic function in AL-CA patients may assist obtaining pre-therapeutic information and improve prognosis. Sharmila et al. indicated that the longitudinal strain, reflecting subclinical LV systolic function, is often reduced in CA patients as compared with hypertensive LV hypertrophy, suggesting that the longitudinal dysfunction is linearly associated with microvascular impairment [9]. Taken together, we hypothesized that coronary microvascular dysfunction is involved in LV systolic impairment and that the evaluation of regional myocardial microcirculation in AL-CA patients may facilitate the early identification of the regional impairment of systolic function.

As significant differences in first by-pass perfusion parameters were observed between the AL-CA patients with impaired or preserved systolic function and the normal controls, these perfusion data may assist in discriminating between the microvascular dysfunction AL-CA patients and normal controls. The data obtained from the ROC analysis supported the use of the upslope, TTM, and MaxSI as criteria to enable the differentiation of the microcirculation of AL-CA patients, regardless of LVEF, from that of normal control individuals. Besides, precise identification of amyloisosis type is also crucial in clinical setting due to the prognosis and the treatment depends on the type of this disease. CMR LGE could help differentiate noninvasively between cardiac light chain amyloid and transthyretin-related amyloidosis (ATTR), and among different ATTR pathogenic mutation groups [29, 38]. As previously research reported, coronary microvascular dysfunction is highly prevalent in cardiac amyloidosis patients regardless of the underlying type of amyloid deposits [9], however, the precise clinical utility of our findings are not clear. Further study is require to determine if myocardial perfusion parameter could help classify the type of cardiac amyloidosis, and may subsequently be an early clinical makers for screening and identification asymptomatic relatives for phenotypic heterogeneity in hereditary ATTR amyloid. In addition, the process of successful treatment of AL amyloidosis aiming to eliminate or control the dyscrasia that produces the amyloid paraprotein and reflecting by a decrease in cardiac biomarkers, may prior to a change in typical morphologic appearances, and whether coronary microvascular function also response to chemotherapy needs further investigation.

Our study had several limitations. Firstly, a larger sample size of AL-CA patients is required for further investigation, with an emphasis on myocardial perfusion. Secondly, our present study did not evaluate whether the interstitial myocardial fibrosis detected by LGE or quantified by T1 blood-pool gadolinium kinetics or T1 mapping on CMR could affect coronary microvascular dysfunction, which are valuable for differential diagnosis and the prediction of outcomes in CA patients [39–41]. The association between LGE, and T1 blood-pool gadolinium kinetics or T1 mapping, and first-perfusion imaging on CMR in CA patients will be investigated in our future study. Thirdly, resting/exercise electrocardiogram, echocardiography and laboratory examination was performed to exclude coronary heart disease, however, not all AL-CA subjects received coronary angiography in our study, which could help to scientifically prove that ischemia is caused by microvascular dysfunction. Finally, patients with LVEF < 50 % were defined as exhibiting impaired systolic function in our study. However, LVEF reflects the global LV systolic function and is less sensitive in assessing the regional systolic function. In addition, CA patients has smaller cavity sizes (reduced LVEDV) due to increased wall thickness could result in a comparative decrease in EF compared with normal controls, EF may be >50 % in patients with small cavity size result from concentric “hypertrophy”, thus may not accurate to evaluated the systolic function and may consequently mis-classifying some patients with truly impaired systolic function into the normal systolic function group. Dan Liu et al. previously reported that longitudinal dysfunction was associated with poor outcome in CA patients with preserved LVEF [42]. Therefore, the association between first-pass data and more sensitive indices reflecting the LV systolic function, including strain and pulsed tissue Doppler echocardiography, which can assist in the early identification of systolic dysfunction, will be discussed in our future study.

## Conclusions

The difference of LV regional myocardial microvascular function among AL-CA patients with or without LV systolic dysfunction, and normal subjects can be monitored by first-pass perfusion CMR imaging, and the threshold values of LV regional myocardial perfusion parameters may be used as indicators for differentiating myocardial microvascular dysfunction between AL-CA patients with preserved or impaired systolic function and normal subjects.
